# Efficacy and Clinical Determinants of Antipsychotic Polypharmacy in Psychotic Patients Experiencing an Acute Relapse and Admitted to Hospital Stay: Results from a Cross-Sectional and a Subsequent Longitudinal Pilot Study

**DOI:** 10.1155/2014/762127

**Published:** 2014-01-27

**Authors:** Felice Iasevoli, Elisabetta F. Buonaguro, Massimo Marconi, Emanuela Di Giovambattista, Maria Paola Rapagnani, Domenico De Berardis, Giovanni Martinotti, Monica Mazza, Raffaele Balletta, Nicola Serroni, Massimo Di Giannantonio, Andrea de Bartolomeis, Alessandro Valchera

**Affiliations:** ^1^Department of Neuroscience, Reproductive Sciences and Odontostomatology, University “Federico II” of Naples, Via Pansini 5, 80131 Naples, Italy; ^2^Hermanas Hospitalarias, Villa San Giuseppe Hospital, 63100 Ascoli Piceno, Italy; ^3^NHS, Department of Mental Health, Psychiatric Service of Diagnosis and Treatment, Hospital “G. Mazzini”, ASL 4, 64100 Teramo, Italy; ^4^Department of Neurosciences and Imaging, University “G. d'Annunzio” of Chieti, 66013 Chieti, Italy; ^5^Department of Life, Health and Environmental Sciences, University of L'Aquila, 67010 L'Aquila, Italy; ^6^FORIPSI, 00199 Rome, Italy

## Abstract

*Background.* Antipsychotic polypharmacy is used in several psychiatric disorders, despite poor evidence existing to support this practice. *Aim.* We evaluated whether psychotic patients in acute relapse exposed to antipsychotic polypharmacy (AP + AP) showed different demographic, clinical, or psychopathological features compared to those exposed to one antipsychotic (AP) and whether AP + AP patients showed significantly higher improvement compared to AP patients after a 4-week treatment. *Methods.* Inpatients were subdivided into AP + AP and AP ones. In the cross-sectional step, patients were compared according to demographics, clinical variables, and scores on rating scales. In the longitudinal step, patients remained for 4 weeks under admission medications and were compared for clinical improvement. *Results.* AP + AP patients were more frequently diagnosed with schizophrenia and mental retardation as a comorbid illness. AP + AP patients were more frequently under first-generation antipsychotics and had worse clinical presentation. After 4 weeks of treatment, both AP + AP and AP patients improved compared to the baseline. However, AP patients scored significantly less than AP + AP patients at the Clinical Global Impression Scale at the 4-week time point but not at the baseline, indicating a treatment-specific improvement. *Conclusions.* Antipsychotic polypharmacy may be offered to specific types of psychotic patients. However, efficacy of this strategy is limited at best.

## 1. Introduction 

Antipsychotic drugs are currently used to treat psychotic symptoms in a wide array of psychopathological conditions. Despite the fact that international guidelines recommend prescribing antipsychotic polypharmacy only as the ultimate step [1, 2], antipsychotic polypharmacy is very common in clinical practice. A recent longitudinal perspective survey in Japanese health institutions has found that up to 20% patients suffering from schizophrenia had been exposed to antipsychotic polypharmacy during the 2-year time-window of the study [3]. In a large Italian population from acute inpatient facilities, antipsychotic polypharmacy was recorded in one third of the patients [4], irrespective of the diagnosis. Overall, variable but yet substantial rates of antipsychotic polypharmacy have been described, depending on the sample composition and setting [5–10]. However, factors predisposing to antipsychotic polypharmacy in general psychiatric population and its actual efficacy have been poorly studied to date. Moreover, little is known on the psychopathological determinants that induce psychiatrists to prescribe an association of antipsychotics in acute relapsing psychotic patients.

It has been stated that antipsychotic polypharmacy may represent a valuable option for some clinically difficult conditions; however, it should be avoided in the majority of patients [11]. A series of uncontrolled small open-label studies have found that different antipsychotic polypharmacy combinations may have favorable outcome compared to previous monotherapy [12–14]. However, the lack of a control group represented a major challenge to generalize the results of these studies.

Despite expert opinions, evidence to support or discourage antipsychotic polypharmacy is limited, representing a clinical unmet need of modern psychiatry. A recent comprehensive meta-analysis has reported that antipsychotic polypharmacy is superior to monotherapy in terms of less inefficacy and all-cause discontinuations [15]. However, presumptive publication bias and heterogeneity of the database do not allow deriving firm clinical recommendations. Another systematic review has only found limited efficacy of antipsychotic polypharmacy in clozapine-resistant schizophrenia patients [16]. However, advantages of antipsychotic polypharmacy were countered by several drawbacks, such as increased mortality, high nonadherence, decreased cognitive functioning, or extra costs [16].

Based on these considerations, we carried out a pilot study that was aimed at (i) dissecting the clinical determinants associated with antipsychotic polypharmacy in acute psychiatric patients; (ii) evaluating whether antipsychotic polypharmacy in these patients may be more efficacious than treatment by one antipsychotic only, at least in the first weeks of treatment.

To achieve these goals, we first compared demographic, clinical, and psychopathological data in acute patients admitted to hospital stay under antipsychotic polypharmacy compared to those admitted with one antipsychotic only. Then, we selected a group of cases (polypharmacy) and a group of matched controls and compared improvements in psychopathological measures at discharge. The results of these clinical trials have been discussed bearing in mind the pilot nature of the study.

## 2. Methods

### 2.1. Patients

The study was conducted at the “Villa San Giuseppe” Hospital of Ascoli Piceno, Italy, during 2012. All inpatients consecutively admitted to this center because of an acute relapse of their primary psychiatric illness were considered eligible. Diagnoses were made by trained psychiatrists using the Structured Clinical Interview for Diagnosis for Axis-I disorders/Patient edition (SCID-I/P, [17]) and for Axis-II disorders [18].

Inclusion criteria were (i) being under antipsychotic treatment, irrespective of the diagnosis; (ii) age between 18 and 65 years. Exclusion criteria were (i) severe neurological disorders; (ii) severe systemic diseases; (iii) psychiatric diagnosis due to general medical condition or to substance abuse, if not in comorbidity with another Axis-I diagnosis; (iv) Axis-II diagnosis, if not in comorbidity with an axis-I diagnosis (other than a psychiatric diagnosis secondary to general medical condition or to substance abuse); (v) a condition of treatment resistance, according to Kane and APA's criteria [1, 19].

Patients were adequately informed of all aspects regarding the participation and the purpose of the study, providing a written informed consent prior to being enrolled. Local Ethical Committee was appropriately informed and approved the study. All procedures carried out in the present study complied with the principles laid down by the World Medical Association Declaration of Helsinki (as amended by the 59th General Assembly, Seoul, Republic of Korea, October 2008).

### 2.2. Study Design

The first part of the study was carried out according to a case-control cross-sectional design. Cases were defined as those patients that were under two or more antipsychotic drugs (AP + AP) at the time of evaluation (i.e., at admission to hospital stay). Controls were those patients under one antipsychotic (AP) at the time of evaluation. In both AP + AP and AP patients, adjunctive psychotropic or nonpsychotropic treatments were allowed and did not determine exclusion from the study.

The second part of the study was carried out according to a case-control 4-week open-label longitudinal design. Cases and controls were chosen among those constituting cases (AP + AP) and controls (AP), respectively, of the first part of the study. Both cases and controls continued antipsychotic and nonantipsychotic treatments prescribed at their admission.

Inclusion and exclusion criteria were those of the cross-sectional study. Physicians were allowed to modify drug doses and types according to clinical conditions. However patients in both groups, whose antipsychotic or antipsychotic dose was changed during the 4 weeks of hospital stay, were dropped from the study. Modifications of nonantipsychotic medications were allowed without causing dropout from the study.

Based on these criteria, only 21 out of the 41 cases included in the cross-sectional study were still eligible for inclusion in the subsequent longitudinal study. Cases were then matched with 21 controls. Care was taken to carefully match cases and controls in terms of baseline demographic and clinical characteristics. Variables that were taken into account for matching were age; gender distribution; age at disease onset; duration of pathology; distribution of axis-I diagnosis; distribution of comorbid diagnosis. Matching did not include distribution of psychotropic agents (either antipsychotics or not); baseline scores on rating scales.

### 2.3. Assessments and Outcomes

The following data were recorded for all patients at their hospital admission: age at first disease diagnosis; duration of pathology (i.e., years from the first diagnosis); current psychotropic and nonpsychotropic agents. Doses of antipsychotic medications received by each patient at the moment of the evaluation were recorded and adjusted in chlorpromazine equivalent doses [20].

At admission, each patient was administered or self-rated the following rating scales: Young Mania Rating Scale (YMRS, [21]); the Italian version of the 24-item BPRS [22]; 21-item Hamilton Scale for Depression (HAM-D, [23]); Hamilton Scale for Anxiety (HAM-A, [24]); Clinical Global Impression-Severity (CGI, [25]); the Italian version of the Barratt Impulsiveness Scale (BIS-11, [26]); Toronto Alexithymia Scale (TAS-20, [27]); 10-item Drug Attitude Inventory (DAI-10, [28]); the Italian version of the Positive and Negative Syndrome Scale (PANSS, [29]).

Patients included in the longitudinal study repeated these measures at the 4-week time-point.

In the cross-sectional study, we evaluated whether significant differences occurred between AP + AP and AP patients in (1) demographic variables; (2) distribution of psychiatric diagnoses and comorbid diagnoses; (3) distribution of psychotropic drugs; (4) antipsychotic doses; (5) scores on rating scales.

In the longitudinal study, we investigated whether significant differences occurred between AP + AP and AP patients in (1) psychotropic drug distribution and antipsychotic doses at the 4-week time-point; (2) symptoms improvement after 4 weeks of treatment compared to admission, in each group; (3) symptoms improvement after 4 weeks in cases versus controls.

### 2.4. Statistics

Statistical analyses were carried out using JMP 9.0 and GRAPHPAD PRISM 5.0 software for Mac. Categorical data were analyzed by *χ* ^2^ test. Two-tailed unpaired Student's *t* and two-way ANOVA tests were used to compare parametric data. In all tests, significance was set at *P* < 0.05.

## 3. Results 

### 3.1. Cross-Sectional Study

We screened a total of 298 patients consecutively admitted to the “Villa San Giuseppe” Hospital during 2012. Among these inpatients, 145 patients (72 males) met inclusion criteria to be enrolled in the study.

Included patients were subdivided into 41 cases (18 males) and 104 controls (54 males). Among these patients, only the following axis-I psychiatric diagnoses were found: schizophrenia (33%), schizoaffective disorder (5.5%), bipolar disorder I (BD-I, 25%), and bipolar disorder not otherwise specified (BD-NOS, 36.5%). Among comorbid diagnoses, alcoholism abuse/dependence (33%), mental retardation (5.5%), cognitive impairment (9%), and personality disorders (13%) were the most frequently represented. Other diagnoses were absent or were observed in no more than one patient in the entire samples, and have not been reported.

AP + AP patients were significantly younger at the time of evaluation and had lower age at disease onset, and longer duration of disease compared to AP patients (Table 1). Distribution of both psychiatric diagnosis and comorbid diagnosis was significantly different between cases and controls (Table 1). AP + AP patients more frequently suffered from schizophrenia than AP patients, while AP patients were significantly more frequently diagnosed with BD-NOS than AP + AP patients. Schizoaffective disorder and BD-I were similarly distributed between cases and controls.

Comorbid diagnosis distribution was significantly different between cases and controls (Table 1). AP + AP patients more frequently suffered from mental retardation compared to AP patients. These latter patients were more frequently diagnosed with personality disorders and cognitive impairment; however the difference between groups was not significant.

Psychotropic agents were also differentially prescribed to cases and controls (Table 1). AP + AP patients were significantly more frequently prescribed first-generation antipsychotics, while AP patients were more frequently exposed to mood stabilizers and benzodiazepines (Table 1). Exposure to second-generation antipsychotics, antidepressants, and anticholinergics was not significantly different between AP + AP and AP patients (Table 1). Moreover, AP + AP patients were prescribed significantly higher daily doses of antipsychotics compared to AP patients (Table 1).

Considering 600 mg/day of chlorpromazine as the threshold for the highest recommended dose [20], 33 (80.5%) out of 41 AP + AP patients were prescribed over-the-threshold antipsychotic doses. Only 6 (5.7%) out of 104 AP patients were prescribed suprathreshold antipsychotic doses.

Among AP + AP patients, adjunctive antipsychotics were at low doses (i.e., ≤150 mg/day of chlorpromazine) in 12 (29%) patients, at full doses (i.e., within therapeutic range) in 20 (49%) patients, and at high doses in 9 (22%) patients.

**Figure 1:**
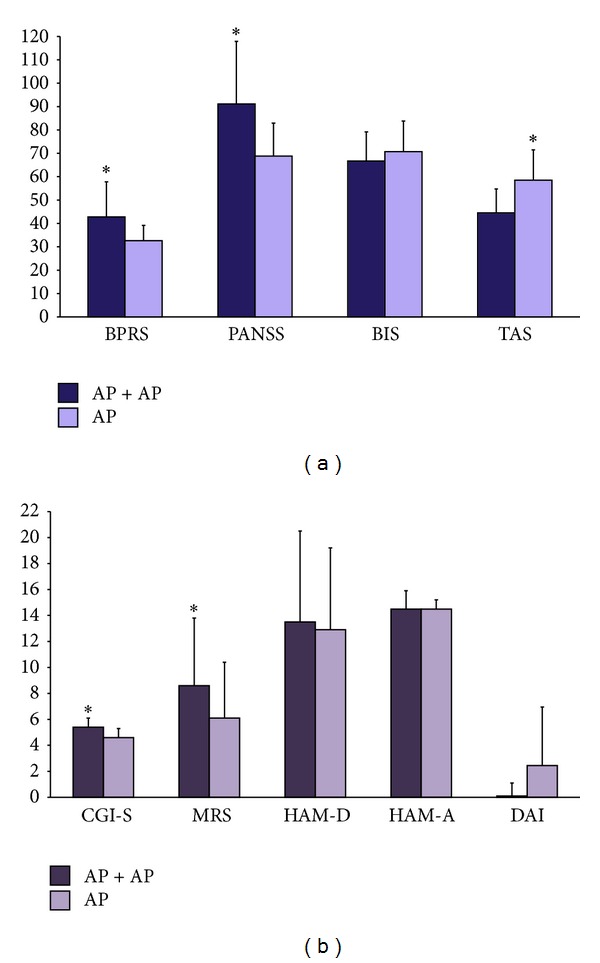
Scores on rating scales in the cross-sectional study. Here are depicted the mean scores ± standard error means on the following rating scales administered to the patients enrolled in the cross-sectional study: the Young Mania Rating Scale (MRS); the 24-item Brief Psychiatric Rating Scale (BPRS); 21-item Hamilton Scale for Depression (HAM-D); Hamilton Scale for Anxiety (HAM-A); Clinical Global Impression-Severity (CGI-S); the Barratt Impulsiveness Scale (BIS); Toronto Alexithymia Scale (TAS); 10-item Drug Attitude Inventory (DAI); the Positive and Negative Syndrome Scale (PANSS). *Significant differences (*P* < 0.05) at the Student's *t* test between AP + AP and AP patients.

Scores on multiple-rating scales were significantly different between cases and controls (Figure 1). AP + AP patients scored significantly higher on the BPRS, the PANSS, the CGI, and the YMRS compared to AP patients (Figure 1). AP patients scored significantly higher than AP + AP ones on the TAS-20. No significant differences were observed on the BIS-11 and the DAI-10 (Figure 1).

### 3.2. Longitudinal Study

Cases (*n* = 25, 12 males) and controls (*n* = 26, 16 males) were carefully matched in order to avoid any significant difference in demographic variables, distribution of axis-I psychiatric diagnosis, and distribution of comorbid diagnosis (Table 2). During the 4-week trial, 4 cases and 5 controls dropped out from the study due to changes in antipsychotic medication or doses.

After matching, we observed a significant difference in the distribution of psychotropic agents between cases and controls (Table 2). Indeed, AP + AP patients were significantly more frequently exposed to first-generation antipsychotics compared to AP patients (Table 2). No significant difference in the prescription of the other psychotropic agents was found between groups (Table 2). AP + AP patients were prescribed significantly higher daily doses of antipsychotics compared to AP patients (Table 2).

We evaluated whether significant symptom improvements occurred at the 4-week time-point in cases compared to controls. To carry out this analysis, we adopted two-way ANOVA in order to evaluate whether significant score changes could depend on the effect of treatment (AP + AP versus AP), on the effect of time (4-week time-point versus baseline), or on the combined effect of treatment *x* time.

**Figure 2:**
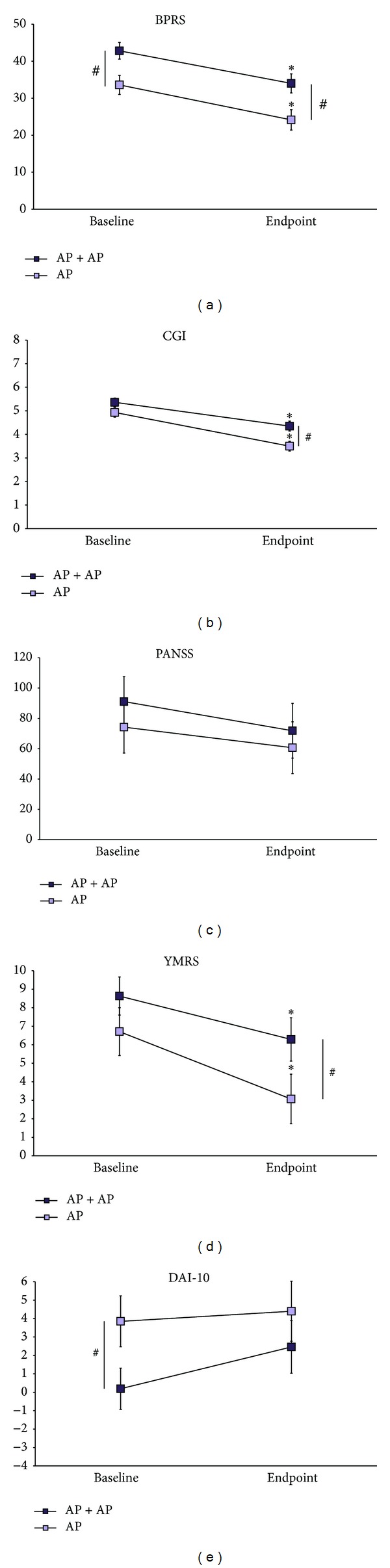
Scores on rating scales in the longitudinal study. Here are depicted the mean scores ± standard error means on the rating scales administered to the patients enrolled in the longitudinal study. By two-way ANOVA, we have compared (i) score differences in AP + AP patients compared to AP patients, at baseline and at endpoint (^#^ *P* < 0.05); (ii) score differences within each group at endpoint versus baseline (*P < 0.05).

BPRS scores were affected by treatment (*P* = 0.0004, df = 3.63, and *t* = 3.73) and by time (*P* = 0.0007, df = 3.63, and *t* = 3.58). No treatment *x* time effect was found (*P* > 0.05, df = 3.63). Both AP + AP and AP patients improved at the 4-week time-point compared to baseline (time effect; Figure 2). AP patients scored significantly lower than AP + AP patients at baseline and at the 4-week time-point (treatment effect; Figure 2).

DAI-10 scores were affected by treatment (*P* = 0.05, df = 3.54, and *t* = 1.99). No time (*P* > 0.05, df = 3.54) or treatment *x* time effect (*P* > 0.05, df = 3.54) was found. No significant improvement was found in both AP + AP and AP patients at the 4-week time-point compared to baseline. AP patients scored significantly higher than AP + AP patients at baseline; however no significant difference was observed at the 4-week time-point (Figure 2).

YMRS and CGI scores were affected by treatment (YMRS: *P* = 0.039, df = 3.62, and *t* = 2.11; CGI: *P* = 0.0025; df = 3.59; *t* = 3.16) and by time (YMRS: *P* = 0.017, df = 3.62, and *t* = 2.45; CGI: *P* < 0.0001; df = 3.59; *t* = 6.01). No treatment *x* time effect was found (YMRS: *P* > 0.05, df = 3.62; CGI: *P* > 0.05, df = 3.59). Both AP + AP and AP patients significantly improved at the 4-week time-point compared to baseline (time effect). Notably, AP patients scored significantly lower than AP + AP patients at the 4-week time-point (treatment effect) but not at baseline (Figure 2).

PANSS scores were not affected by time (*P* > 0.05, df = 3.32), by treatment (*P* > 0.05, df = 3.32), or by treatment *x* time (*P* > 0.05, df = 3.32). Both AP + AP and AP patients did not significantly improve on PANSS scores at the 4-week time-point compared to baseline (Figure 2). No significant differences on PANSS scores were found between cases and controls at baseline and at the 4-week time-point (Figure 2).

## 4. Discussion

The main results of our study were that (i) patients exposed to antipsychotic polypharmacy differ from patients exposed to one antipsychotic in many demographic and clinical respects; (ii) treatment with two or more antipsychotics is associated with questionable clinical improvement compared to treatment with one antipsychotic. These main findings should be considered in the light of the pilot nature of our study.

Patients exposed to antipsychotic polypharmacy were more frequently diagnosed with schizophrenia on axis-I and mental retardation as a comorbid diagnosis compared to those not exposed to antipsychotic polypharmacy. Conversely, these latter patients were more frequently diagnosed with bipolar disorder. The finding that schizophrenia patients are more likely to receive combinations of antipsychotics is not surprising and is in line with previous reports [30]. Schizophrenia patients may be more difficult to treat with one antipsychotic only for a number of reasons, for example, refractory psychotic symptoms, refractory nonpsychotic symptoms, and loss of efficacy of the first antipsychotic [30]. As well, individuals suffering from mental retardation syndromes have been found poorly responsive to antipsychotic treatments [31].

In these conditions, augmentation of the first antipsychotic with a second one (either at full recommended or at low dose) is supposed to help gaining or recovering full clinical efficacy.

According to this view, here we have observed that patients under antipsychotic polypharmacy are more frequently prescribed first-generation antipsychotics and less frequently prescribed benzodiazepines compared to those under one antipsychotic only. Moreover, in approximately one-third of the patients under antipsychotic polypharmacy, the adjunctive antipsychotic (almost constantly a first-generation one) is given at low doses.

These findings may suggest that (i) antipsychotic augmentation may be more likely provided by a first-generation antipsychotic; (ii) the first-generation antipsychotic may be used at low doses to impact residual psychotic and nonpsychotic symptoms, such as agitation, anxiety, or insomnia.

It has been suggested that some schizophrenia patients may suffer from a deficit syndrome, whose clinical characteristics include younger age at onset, chronic course, and poor response to antipsychotics [32]. In our sample, patients exposed to antipsychotic polypharmacy were younger and had younger age at onset and longer duration of pathology compared to those under one antipsychotic only. These features may account for a more chronic and severe course of the disease. Notably, our results are consistent with those described in a recent nation-wide study on the Finnish population, reporting higher rates of antipsychotic polypharmacy in those schizophrenia patients with longer duration of disease [33].

It can be hypothesized that antipsychotic polypharmacy may be offered to those patients with more chronic course and suffering from deficit schizophrenia. Intriguingly, deficit schizophrenia has some common features with mental retardation. The chronic course of the above-mentioned diseases may expose patients to prolonged antipsychotic treatments, thereby favoring the loss of antipsychotic efficacy [34] and inducing physicians to adopt antipsychotic polypharmacy strategies.

However, some criticism has been raised on the actual advantages of antipsychotic polypharmacy [8, 16]. In the second part of the study, we investigated clinical efficacy of antipsychotic polypharmacy in a matched case-control design.

We observed that both groups of patients (i.e. those under antipsychotic polypharmacy and those under one antipsychotic only) significantly improved in global psychopathology after 4 weeks of treatment. However, patients under one antipsychotic improved significantly more than those under antipsychotic polypharmacy at the CGI scale.

These observations should pose severe doubts about the rationale of exposing a patient to antipsychotic polypharmacy. This consideration holds to be more true since there is consistent evidence that antipsychotic polypharmacy causes high rates of side effects [16]. It appears that polypharmacy may be as useful as monotherapy in controlling psychotic symptoms, but it may be less advantageous than monotherapy in improving the global psychopathological condition of a patient.

Due to the pilot nature of this study, however, trials with larger samples and more stringent stratification criteria are needed to expand the results discussed herein.

This study has several limitations that should be taken into account when discussing results. Sample size was relatively small in the first and in the second step of the study. Despite being relatively common, however, patients under antipsychotic polypharmacy are still a minor part of patients undergoing antipsychotic medications. This consideration may explain, at least in part, the difficulty to gain larger sample size relatively to the patients under investigation in this study.

The small sample size also prevented controlling for covariates in the analysis of the determinants of antipsychotic polytherapy, not allowing to carry out a multivariate analysis. The case should be that some factors positively associated with polytherapy might disappear when controlling for covariates. Despite this limitation, the results in this study should be considered preliminary data prompting more accurate evaluations in future trials.

Antipsychotic polypharmacy has been studied as a whole, without differentiating between specific combinations of antipsychotics. It appears that some combinations with specific antipsychotics (e.g., aripiprazole and clozapine) may have a more solid rationale compared to others [35].

No stratification for antipsychotic doses has been carried out, given the low cell size. However, it may be that efficacy outcome may be different when associating two (or more) antipsychotics at full doses compared to the association of an antipsychotic at full dose with another at low dose (e.g., to treat minor residual symptoms).

Antipsychotic therapy has been evaluated in association with other psychotropic and nonpsychotropic medications. Although number and distribution of adjunctive medications have not been found significantly different, it could not be excluded that adjunctive medications may affect response to antipsychotics and therefore may introduce a bias in the study.

The longitudinal study lasted for 4 weeks. This time window has been considered adequate to observe the onset of antipsychotic efficacy and to evaluate whether the patient responds or not to the antipsychotic agent [1, 36]. Nonetheless, response to antipsychotic polypharmacy may improve (or even worsen) after longer period of treatment.

Patients were not randomized to receive either antipsychotic polypharmacy or one antipsychotic only. Randomization was avoided for ethical issues and to follow a naturalistic design that may more closely match clinical reality. However, lack of randomization may introduce another bias in the evaluation of results.

In conclusion, the results of this pilot study provide evidence that antipsychotic polypharmacy may be mainly offered to a subgroup of psychotic patients. However, the actual efficacy of this therapeutic strategy may be limited.

## Figures and Tables

**Table 1:** Patients' demographics and clinical variables in the cross-sectional study.

	AP + AP	AP	*P*	Effect size	df
**Age (years)**	**46.3 ± 11.5**	51.2 ± 14.1*	***0.048***	*t*: **1.99**	**143**
Gender m/f	18/23	54/50	0.76		1
**Age at onset (years)**	**21.6 ± 7.5**	33.5 ± 14.5*	**0.0001**	*t*: **4.14**	**114**
**Duration of disease (years)**	22.8 ± 8.2*	**18.1 ± 11.6**	**0.046**	*t*: **2.02**	**117**
Diagnosis *n* (%)					
(i) **Schizophrenia**	25 (63.4%)*	**23 (22.3%)**	**0.00003**	*x*: **23.44**	**3**
(ii) Schizoaffective	6 (5.8%)	2 (5%)			
(iii) Bipolar disorder type I	7 (18%)	29 (29%)			
(iv) **Bipolar disorder NOS**	**5 (12.8%)**	45 (43.7%)*			
Comorbid diagnosis (*n*):					
(i) **Mental retardation**	5 (31.2%)*	**3 (4.3%)**	**0.0013**	*x*: **13.18**	**3**
(ii) Cognitive impairment	0 (0%)	13 (18.8%)			
(iii) Alcoholism	10 (62.5%)	35 (50.7%)			
(iv) Personality disorder	1 (6.2%)	18 (26.1%)			
Psychotropic drugs *n* (%)					
(i) **First generation antipsychotics**	46 (35.1%)*	**4 (1.8%)**	**<0.00001**	*x*: **83.63**	**5**
(ii) Second generation antipsychotics	43 (32.8%)	81 (37.8%)			
(iii) **Mood stabilizers**	**26 (19.8%)**	85 (39.7%)*			
(iv) Antidepressants	13 (9.9%)	16 (7.5%)			
(v) **Benzodiazepines**	**3 (2.3%)**	28 (13.1%)*			
(vi) Anticholinergics	3 (2.3%)	2 (0.9%)			
**Antipsychotic doses (in mg/day chlorpromazine equivalents)**	954.46 ± 405.71*	**328.72 ± 180.12**	**<0.00001**	*t*: **11.96**	143

In this table we summarized data on (i) demographics in patients receiving antipsychotics polytherapy (AP + AP) compared to patients receiving one antipsychotic only (AP); (ii) rates and distribution of psychiatric and organic comorbid diagnoses in AP + AP versus AP patients; (iii) rates and distribution of antipsychotic and non-antipsychotic psychotropic agents in AP + AP versus AP patients; (iv) antipsychotic doses (in mean mg/day chlorpromazine equivalents) in AP + AP versus AP patients. Demographic and pharmacological variables were compared by Student's *t* test. Diagnosis and antipsychotic distribution were compared by chi-square test. Significant values have been marked in bold and with an asterisk and given with effect size (*t*, *x*). The bold italic items refer to the category where significant differences between groups have been found. df: degrees of freedom.

**Table 2:** Patients' demographics and clinical variables in the longitudinal study.

	AP + AP	AP	*P*	Effect size	df
Age (years)	44.9 ± 10.2	48.2 ± 13.5	0.38		40
Gender m/f	9/12	13/8	0.35		1
Age at onset (years)	24.1 ± 9.4	29.6 ± 13.5	0.19		29
Duration of disease (years)	21.1 ± 7.4	21.1 ± 12.1	0.97		29
Diagnosis *n* (%)					
(i) Schizophrenia	9	17	0.1		3
(ii) Schizoaffective	1	1			
(iii) Bipolar disorder type I	6	2			
(iv) Bipolar disorder NOS	4	1			
Comorbid diagnosis (*n*):					
(i) Mental retardation	2	2	0.15		3
(ii) Cognitive impairment	0	2			
(iii) Alcoholism	5	3			
(iv) Personality disorder	0	1			
Psychotropic drugs *n* (%)					
(i) **First generation antipsychotics**	21*	**1**	**0.002**	*x*: **16.04**	**4**
(ii) Second generation antipsychotics	24	14			
(iii) Mood stabilizers	15	18			
(iv) Antidepressants	2	2			
(v) Benzodiazepines	8	9			
(vi) Anticholinergics	1	2			
**Antipsychotic doses (in mg/day chlorpromazine equivalents)**	920.57 ± 400.09*	**450.66 ± 250.41**	**0.0001**	*t*: **4.31**	**40**

Here, we reported data on (i) demographics in AP + AP versus AP patients included in the longitudinal study; (ii) rates and distribution of psychiatric and organic comorbid diagnoses in AP + AP versus AP patients; (iii) rates and distribution of antipsychotic and non-antipsychotic psychotropic agents in AP + AP versus AP patients; (iv) antipsychotic doses (in mean mg/day chlorpromazine equivalents) in AP + AP versus AP patients. Demographic and pharmacological variables were compared by Student's *t* test. Diagnosis and antipsychotic distribution were compared by chi-square test. Significant values have been marked in bold and with an asterisk and given with effect size (*t*, *x*). df: degrees of freedom.
